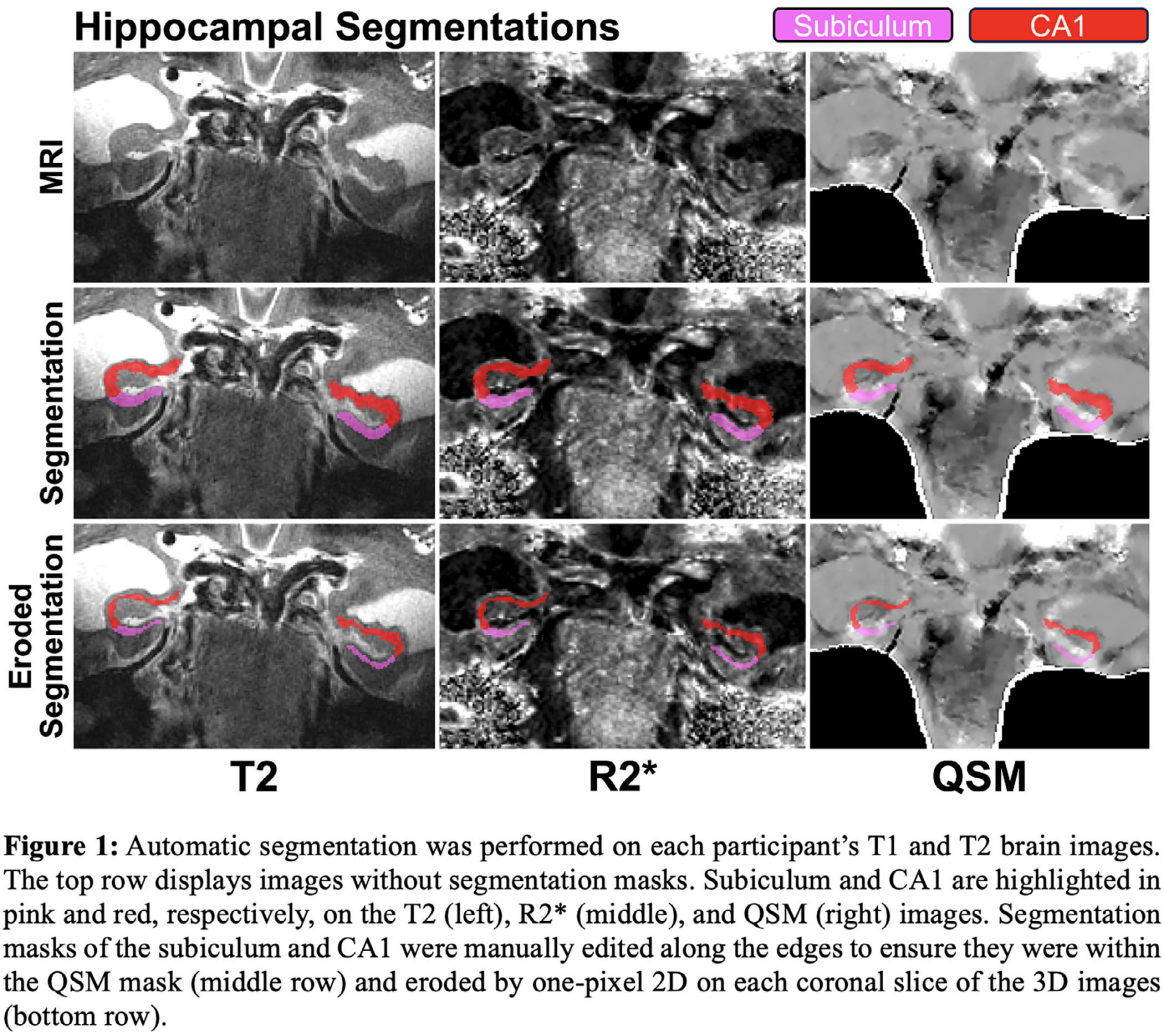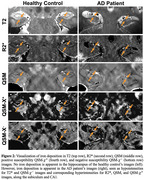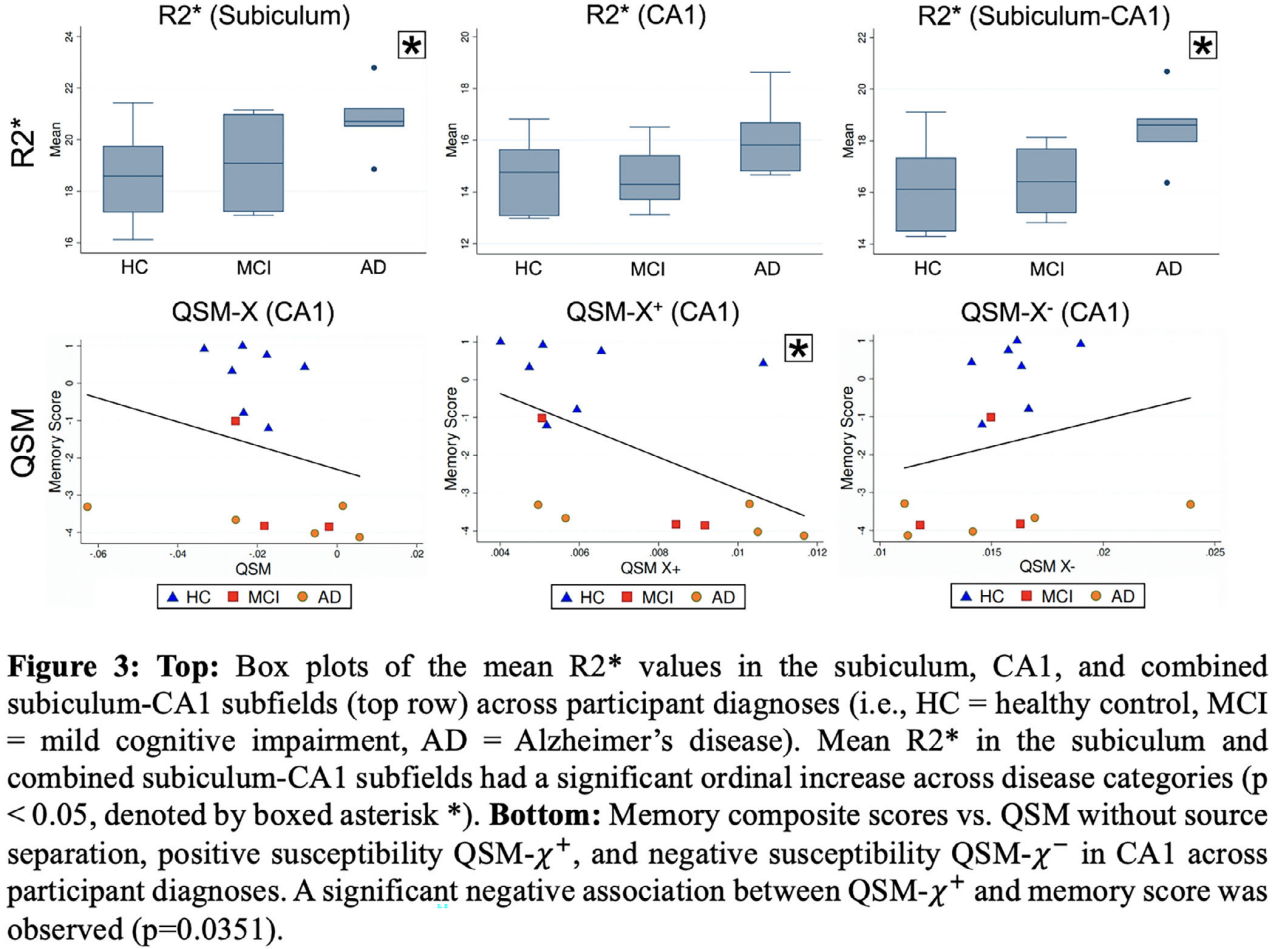# Detecting Hippocampal Subfield Iron in Alzheimer's Disease using Ultra‐high Resolution *in vivo* 7T MRI

**DOI:** 10.1002/alz70856_106116

**Published:** 2026-01-09

**Authors:** Reese A. Dunne, Hossein Moein Taghavi, Phillip DiGiacomo, Julian Maclaren, Meghan Bell, Mackenzie L. Carlson, Elizabeth C. Mormino, Victor W. Henderson, Pascal Spincemaille, Hangwei Zhuang, Yi Wang, Brian S. Rutt, Marios Georgiadis, Michael Zeineh

**Affiliations:** ^1^ Stanford University, Stanford, CA, USA; ^2^ Weill Cornell Medicine, Ithaca, NY, USA

## Abstract

**Background:**

Hippocampal iron, as measured with imaging, biofluids, and histology, has been associated with Alzheimer's disease (AD), its progression, and potentially neuroinflammatory disease mechanisms. With its high sensitivity to tissue magnetic susceptibility, 7T MRI offers the potential to detect abnormal iron deposition within the hippocampus of AD and mild cognitive impairment (MCI) brains *in vivo*, especially when combined with dedicated methods such as quantitative susceptibility mapping (QSM). We aim to utilize ultra‐high resolution 7T MRI and explore conventional and novel source‐separated QSM to quantify hippocampal iron deposition in AD, providing insights into the involvement of brain iron in disease progression.

**Method:**

We conducted 7T MRI on 19 ADRC human volunteers, including 8 healthy controls (HC), 6 individuals with MCI, and 5 with AD. MR images were acquired using a GE MR950 scanner utilizing optical prospective motion correction. Automatic Segmentation of Hippocampal Subfields generated segmentations of the subiculum and CA1 (Figure 1), which were manually edited in a diagnosis‐blind manner, followed by one‐pixel erosion. R2* and source‐separated QSM (positive susceptibility sources QSM‐χ^+^, negative QSM‐χ^‐^) were computed using MEDI and averaged within the subiculum and CA1. Blinded image quality assessments were conducted. Memory composite scores were correlated with iron measurements available in 18 participants. Nonparametric tests quantified hyperintensity gradation in hippocampal QSM/R2* images and assessed the relationship between memory scores and QSM/R2*.

**Result:**

We found a significant ordinal increase of R2* according to participant diagnoses (AD>MCI>HC) in the subiculum (*p* = 0.0445) and combined subiculum‐CA1 (*p* = 0.0232) subfields (examples of negative and positive findings in Figure 2, boxplots in Figure 3‐top), suggestive of increased iron. No significant differences were seen in QSM without source separation, QSM‐χ^+^, or QSM‐χ^‐^. However, a significant negative association between memory scores and QSM‐χ^+^ was observed in CA1 (*p* = 0.0351, Figure 3‐bottom).

**Conclusion:**

We found elevated iron in the subiculum‐CA1 hippocampal subregions *in vivo* in MCI and AD using 7T MRI, correlating with degraded memory performance. Our noninvasive visualization of microscopic hippocampal iron deposition utilizing ultra‐high resolution 7T MRI *in vivo* corroborates post‐mortem data. This translational finding could serve as a novel neuroimaging biomarker for iron‐based AD pathology and inflammation.